# Miscellaneous

**Published:** 1887-12

**Authors:** 


### MISCELLANEOUS.

The Way to Drink.—Here is a plan : Start a hotel or saloon in your own house; be the only customer and you will have no license to pay. Go to your wife and give her $2 to buy a gallon of whiskey, and remember there are sixty drinks in a gallon. You make it your business every time you are thirsty, to buy a drink from your wife, and by the time you will have drank up the first gallon, your wife will have the $2 to pay you back, $2 to put in the bank, and $2 to start business again. And should you happen to live ten years and continue to buy booze from your wife, and die at the expiration of that time, with the snakes in your socks, she will have money enough to bury you decently, buy a good house and lot, educate your children, and marry some respectable man, and never be bothered thinking about you. Try it and see how the thing will pan out.—*Southern Progress.*

Important Decision upon the Matter of Adulterated Food.—A decision has just been made by the Court of Appeals, in the case of the people against Charles Kibler, of Buffalo, of great importance. The defendant was indicted for selling milk adulterated with water, in violation of law. The people proved that the milk sold by the defendant did not reach the standard of purity required by the statute. The defence was that Kibler had bought the milk from a wholesale dealer, and supposed that it was pure ; that he had acted in good faith, etc. The question before the Court of Appeals was, whether a man could be convicted of a criminal charge when he believed he was doing nothing wrong. This is the first time this question has ever been raised in the Court of Appeals of this State, and by affirmance of the judgment of the General Term of the Supreme Court, the Court of Appeals has established the law of the State to be, that the question of knowledge and intent is no defence to a charge of violation of a statute ; that a person who sells milk and butter must know what he is selling. Another important thing about this decision is that if the rule had been established against the people in this case, they would have to show knowledge on the part of a seller of the adulterated article, and it would virtually defeat the object of legislation on the subject, and the adulteration of food could be carried on with impunity. The decision covers the whole question of adulterated food, and it behooves all dealers to know what they are selling.

Where is Heaven ?—The question, "Where is heaven ?" was put to Sam Jones by one of his wealthy church members in Georgia, whose cotton crop yielded him some $20,000 the last year. "Where is heaven ?" said the rich planter. "I'll tell you where heaven is," said Mr. Jones, "If you will go down to the village and buy fifty dollars' worth of groceries, put them in a wagon, and take them to that poor widow on the hillside, who has three of her children sick. She is poor and needs help. Take with you a nurse, and some one to cook their meals. When you get there read the twenty-third psalm and kneel by her side and pray. Then you will find out where heaven is." Next day, as the evangelist was walking through the village, he met this same wealthy planter, his face beaming with joy. He spoke after this manner: "Mr. Jones, I've found out where heaven is. I went as you directed me. We took up the wagon load of groceries, and the poor widow was completely overcome with joy. She could not express her thankfulness. As I read to her the twenty-third psalm, my heart was filled with thankfulness to God; and, when I prayed, the angels came, and I thought I was nearer to heaven than I ever had been in my life. I left the nurse and cook in her humble dwelling, and promised her she should never suffer so long as I could help her."

Dr. W. H. Morse, the author of the text book on "New Therapeutic Agents," says, there is no question in my mind as to the efficacy of Winchester's Hypophosphites, in curing and preventing Pulmonary Consumption ; as their administration supplies the system with oxydizable phosphorus, the deficiency of which, is the proximate cause of the disease. A persistent use of this remedy will abolish the greatest scourge of humanity. It is excellent for general debility and for weak, puny children.

The curious fact that the usual heat produced by friction is absent when articles are magnetized is just now being discussed by scientists who are seeking an explanation. Very striking examples are described in a late number of a scientific periodical. A workman fastened a couple of powerful magnets to his lathe to hold more securely a piece of metal which he wished to drill and turn. The presence of the magnets kept the metal so cold that no water was needed to keep the drill moist and cool. This unusual circumstance may lead to important mechanical advantages. It is such circumstances as the one noted above that lead to valuable discoveries. The scientists, who are looking for a reason why the heat should be absent may not hit upon any valuable idea, but some practical mechanic probably will.

